# Pathway-based molecular subtyping identifies LAMP5 as an EMT-associated prognostic target in colorectal cancer

**DOI:** 10.3389/fphar.2026.1867118

**Published:** 2026-08-19

**Authors:** Xiaoming Gu, Zihan Zhao, Weitang Yuan

**Affiliations:** Department of Colorectal Surgery, The First Affiliated Hospital of Zhengzhou University, Zhengzhou, Henan, China

**Keywords:** colorectal cancer, EMT, LAMP5, molecular subtyping, pathway analysis, single-cell RNA sequencing

## Abstract

**Background:**

Colorectal cancer (CRC) is a highly heterogeneous malignancy with substantial variability in clinical outcomes. Although established molecular classification systems have improved CRC stratification, pathway-level subtyping remains underexplored. A framework integrating pathway-based molecular subtype discovery, multi-cohort validation, single-cell projection, and mechanistic target identification is still lacking.

**Methods:**

Transcriptomic data from TCGA CRC samples were transformed into pathway activity profiles using gene set variation analysis (GSVA), followed by consensus clustering to define pathway-based molecular subtypes. Nearest template prediction (NTP) was applied across 17 independent cohorts to validate subtype robustness. Three CRC single-cell RNA-seq datasets were integrated and analyzed using the single-cell phenotype-associated subpopulation identifier (scPAS) to map subtype-associated signals at cellular resolution. Multi-cohort Cox regression identified prognostic genes, and LAMP5 was further assessed through clinicopathological correlation, pathway enrichment analyses, and experimental validation *in vitro* and *in vivo*.

**Results:**

Four pathway-based molecular subtypes were identified, among which C4 had the worst prognosis. The subtype classification was reproducible across 17 external cohorts. Single-cell analysis suggested that the aggressive subtype-associated phenotype could be projected onto distinct cell populations. LAMP5 emerged as the most consistent unfavorable prognostic gene across cohorts and was associated with advanced clinicopathological features. Functional analyses linked LAMP5 to TGFβ signaling, epithelial-mesenchymal transition (EMT), angiogenesis, invasion, and metastasis. Experimental results supported the pro-tumorigenic role of LAMP5 in CRC.

**Conclusion:**

This study establishes a pathway-based molecular classification framework for CRC and identifies LAMP5 as a robust prognostic biomarker and candidate therapeutic target associated with the EMT pathway.

## 1 Introduction

Colorectal cancer (CRC) remains one of the most common malignancies and a leading cause of cancer-related death worldwide (Bray et al., 2024; Siegel et al., 2024). Despite advances in surgery, chemotherapy, targeted therapy, and immunotherapy, the prognosis of CRC remains highly variable. Patients with similar clinicopathological stage often show markedly different clinical outcomes, suggesting that conventional staging systems do not fully capture the biological heterogeneity of this disease (Hänzelmann et al., 2013; Guinney et al., 2015). A more refined molecular framework is therefore needed to improve prognostic stratification and support precision treatment.

Several transcriptome-based classification systems have substantially advanced the understanding of CRC heterogeneity. The consensus molecular subtype (CMS) framework classifies CRC into four biologically and clinically distinct groups, whereas the CRC intrinsic subtype (CRIS) system focuses on cancer cell-intrinsic transcriptional features (Isella et al., 2017). These frameworks have provided important insights into stromal composition, immune contexture, and subtype-associated prognosis. However, gene-level classification remains susceptible to transcriptional noise, platform variation, and the confounding effects of mixed cellular components in tumor tissues (Malla et al., 2024). Pathway-level analysis offers a complementary strategy by converting gene expression data into coordinated biological activity profiles. Gene set variation analysis (GSVA), for example, quantifies pathway activity at the individual-sample level and may provide a more stable and functionally interpretable representation of tumor heterogeneity (Li et al., 2025). Recent work has further suggested that pathway-centered stratification can refine subtype definition and uncover biologically meaningful states in CRC (Liu et al., 2024).

Single-cell RNA sequencing (scRNA-seq) has further expanded the understanding of CRC by revealing the cellular diversity of the tumor microenvironment and the interactions among epithelial, immune, and stromal compartments (Schwake et al., 2013; Umeda et al., 2022; Xie et al., 2024; Zhang et al., 2025b). These studies indicate that malignant progression is shaped not only by cancer cell-intrinsic programs but also by microenvironmental remodeling and lineage-specific cellular states. However, most molecular subtype models are established using bulk transcriptomic data, and their biological significance at single-cell resolution often remains unclear. Bridging cohort-level molecular phenotypes with individual cellular populations is therefore an important step toward mechanistic interpretation. The recently developed single-cell phenotype-associated subpopulation identifier (scPAS) provides a useful framework for projecting phenotype-associated signatures derived from bulk transcriptomic data onto single-cell datasets, thereby enabling the identification of cellular subsets associated with aggressive molecular programs (Xie et al., 2024).

Lysosome-associated membrane protein 5 (LAMP5) is a member of the lysosome-associated membrane protein family, whose members are involved in membrane trafficking, lysosomal function, and cancer-related biological processes (Martinez-Romero et al., 2018). Although LAMP5 has been less extensively studied than other family members, emerging evidence suggests that it may contribute to tumor progression in several malignancies. Previous studies have linked LAMP5 to leukemogenesis, metastatic potential in gastric cancer, and oncogenic or immunological features in pan-cancer settings (Schubert et al., 2018; Tao et al., 2024; Tang et al., 2026). Nevertheless, its role in CRC remains poorly defined, particularly regarding molecular subtype heterogeneity, prognosis, and signaling mechanisms associated with malignant progression. In the present study, we established a pathway-based molecular classification framework for CRC, validated the identified subtypes across multiple independent cohorts and projected aggressive subtype-associated signals at single-cell resolution, and screened for core prognostic genes associated with the aggressive subtype. We further identified LAMP5 as a robust unfavorable prognostic biomarker and investigated its association with TGFβ-EMT signaling and CRC progression through integrated bioinformatics analyses and experimental validation.

## 2 Materials and methods

### 2.1 Data acquisition and preprocessing

RNA sequencing data and corresponding clinical information for CRC were obtained from The Cancer Genome Atlas (TCGA), including colon adenocarcinoma (TCGA-COAD) and rectum adenocarcinoma (TCGA-READ) cohorts. Publicly available Gene Expression Omnibus (GEO) datasets were used for external validation, including TCGA_CRC, GSE39582, GSE161158, GSE41258, GSE87211, GSE39084, GSE28702, GSE26682, GSE17537, GSE13294, GSE12945, GSE72970, GSE38832, GSE71187, GSE28722, GSE17536, and GSE14333. Additional datasets, including GSE31595, GSE143985, GSE103479, GSE106584, and GSE29621, were used for clinicopathological correlation and prognostic evaluation where applicable. scRNA-seq datasets were obtained from GEO under accession numbers GSE236698, GSE188711, and GSE166555. As only publicly available datasets were analyzed, no additional ethical approval was required for the bioinformatics component of this study.

### 2.2 Pathway activity quantification

To characterize pathway-level activity in CRC, GSVA was performed on the TCGA CRC transcriptomic matrix using the GSVA R package (version 1.50.1). Gene sets were retrieved from the Molecular Signatures Database (MSigDB), including Gene Ontology (GO), Kyoto Encyclopedia of Genes and Genomes (KEGG), and Hallmark collections. Enrichment scores were calculated for each sample to generate a pathway activity matrix, which was subsequently used for pathway-based molecular subtype discovery.

### 2.3 Consensus clustering for pathway-based molecular subtype identification

Consensus clustering was performed on the pathway activity matrix using the ConsensusClusterPlus R package (version 1.66.0). The clustering procedure was conducted with the following parameters: reps = 1000, pItem = 0.8, pFeature = 1, distance = “euclidean”, clusterAlg = “hc”, and innerLinkage = “ward. D2”. Candidate cluster numbers were evaluated using cumulative distribution function (CDF) curves, delta area plots, and the proportion of ambiguous clustering (PAC). Based on these criteria, the optimal number of clusters was determined to be four. Principal component analysis (PCA), uniform manifold approximation and projection (UMAP), and t-distributed stochastic neighbor embedding (t-SNE) were used to visualize subtype separation. Survival differences among subtypes were assessed using Kaplan-Meier analysis with the log-rank test.

### 2.4 Nearest template prediction analysis

To validate the reproducibility of the identified pathway-based molecular subtypes, nearest template prediction (NTP) was applied across 17 independent CRC cohorts. Subtype-discriminative template genes were identified in the TCGA discovery cohort by differential expression analysis comparing each subtype against all remaining samples (one-versus-rest comparisons). Genes were ranked by the magnitude and statistical significance of their subtype-specific upregulation, and the top 300 most discriminative genes across the four subtypes (Benjamini–Hochberg adjusted P < 0.05) were retained as template features. Each external dataset was independently assigned to one of the four subtypes (C1-C4) according to similarity to the subtype templates. This strategy was used to assess the transferability and robustness of the subtype framework across cohorts.

### 2.5 Functional enrichment analysis

To characterize the biological features of each pathway-based molecular subtype, Gene Ontology biological process enrichment analysis was performed using the clusterProfiler R package. Genes characteristic of each subtype were subjected to enrichment analysis, and terms with adjusted P values <0.05 were considered statistically significant. The top enriched terms for each subtype were used for downstream interpretation and visualization.

### 2.6 Single-cell RNA-seq data processing and integration

scRNA-seq datasets from GSE236698, GSE188711, and GSE166555 were processed using the Seurat R package (version 4.4). Standard quality control procedures were applied to remove low-quality cells and technical outliers according to conventional metrics, including the number of detected genes and the proportion of mitochondrial transcripts. To reduce batch effects while preserving biological variation, the three datasets were integrated using the Harmony algorithm. Following dimensionality reduction, unsupervised clustering was performed at a resolution of 1. Cell clusters were manually annotated according to canonical marker genes, resulting in 11 major cell populations, including B cells, fibroblasts, T/NK cells, mural cells, endothelial cells, monocytes, plasma cells, epithelial cells, macrophages, dendritic cells, and mast cells.

### 2.7 Single-cell pathway activity scoring

To project the aggressive pathway-based molecular subtype to single-cell resolution, the top 300 genes associated with the C4 subtype were used as the input signature for AUCell-based scoring. A single-cell pathway activity score was calculated for each cell, and the observed score was compared with a null distribution generated by permutation to obtain a normalized phenotype-association score and an associated significance value for every cell. Cells whose scores were significantly positively associated with the aggressive C4 phenotype (Benjamini–Hochberg adjusted P < 0.05) were defined as scPAS+, cells significantly negatively associated were defined as scPAS-, and the remaining cells were defined as neutral. This procedure was used to identify cellular populations enriched for the aggressive bulk-derived phenotype. Downstream comparisons were then performed between scPAS+ and scPAS- populations to evaluate functional differences.

### 2.8 Pathway and hallmark enrichment analyses

To investigate the signaling context associated with LAMP5, pathway activity inference was performed using the Pathway RespOnsive GENes (PROGENy) R package (version 1.32.0). Pearson correlation analysis was used to evaluate the association between LAMP5 expression and inferred pathway activity scores. Gene set enrichment analysis (GSEA) was conducted by ranking genes according to their correlation with LAMP5 expression and testing the ranked list against Hallmark gene sets. In addition, single-sample gene set enrichment analysis (ssGSEA) was performed using the GSVA package (version 2.4.6) to estimate per-sample hallmark activity scores, including angiogenesis, invasion, epithelial-mesenchymal transition (EMT), and metastasis-related programs. Correlations between LAMP5 expression and these hallmark scores were subsequently evaluated.

### 2.9 Cell culture and gene silencing

Human colorectal cancer cell lines SW480 (Procell, Hubei, China) and HCT116 (Procell, Hubei, China) were cultured in DMEM medium (Gibco, Massachusetts, USA) with 10% fetal bovine serum (Biochannel, Nanjing, China) and 1% penicillin-streptomycin (Servicebio, Wuhan, China). All cells were cultured at 37 °C with 5% CO2. SW480 and HCT116 were selected as two independent and molecularly distinct CRC cell lines: SW480 is a chromosomally unstable, poorly differentiated, mesenchymal-like line with relatively high endogenous LAMP5 expression, whereas HCT116 is a microsatellite-instable (MSI) line representing a different CRC molecular background. Both lines are widely used in CRC migration, invasion, and EMT studies. Using these two complementary models allowed us to assess whether the functional effects of LAMP5 knockdown were consistently observed across different cellular contexts, thereby improving the reliability of the functional validation. To knock down the LAMP5 gene in human colorectal cancer cells, the human shLAMP5 gene sequence was cloned into the lentiviral vector pLKO.1-EGFP-Puro and packaged into lentiviral particles. Empty vectors were used as controls. After infecting cells with lentiviral particles, stable LAMP5-knockdown CRC cell lines were successfully established through 2 weeks of puromycin screening. The knockdown effect was evaluated by quantitative PCR analysis.

### 2.10 Cell proliferation, migration, and invasion assays

Cell proliferation was assessed using the Cell Counting Kit-8 (Solarbio, Beijing, China), following the manufacturer’s guidelines. For analysis, three random microscopic fields were selected from each well and examined using ImageJ software. All procedures were repeated in triplicate. For migration assays, 2 × 10^4^ transfected human colorectal cancer cell lines SW480 (Procell, Hubei, China) and HCT116 (Procell, Hubei, China) were inoculated into the upper chamber of Transwell chambers (Corning, New York, USA) without Matrigel, while 500 μL of complete medium was added to the lower chamber. The migrating cells were fixed with 4% polyformaldehyde (Beyotime, Shanghai, China), stained with crystal violet (Solarbio, Beijing, China), and imaged under an optical microscope (Cewei, Shanghai, China). The cell counts were quantitatively analyzed using ImageJ software. For the wound healing assay, treated and control cells were cultured in 6-well plates containing 10% FBS medium until full confluence. A 200 µL pipette tip was used to create a scratch in the cell monolayer, followed by washing with PBS to remove debris. Images were captured at 0 h and 24 h under an optical microscope and analyzed using ImageJ software to measure wound closure.

### 2.11 Immunohistochemistry

The tumor specimens were fixed with 4% paraformaldehyde buffer overnight and paraffin-embedded and cut into 4 µm sections. Then the sections were deparaffinized three times with xylene and rehydrated in gradient ethanol. Antigen retrieval was performed by microwave heating in EDTA buffer (pH 9.0; Servicebio, Wuhan, China) at 100 °C for 25 min. Endogenous peroxidase activity was blocked with 3% H_2_O_2_ for 30 min, and nonspecific binding sites were blocked using 10% goat serum for 1 h. Sections were incubated with appropriately diluted primary antibodies overnight at 4 °C, followed by incubation with secondary antibodies at room temperature for 1 h. Visualization was achieved using DAB (Servicebio, Wuhan, China), followed by counterstaining with hematoxylin. Bright-field images were captured using an ECLIPSE E100 microscope (Nikon, Tokyo, Japan), and digital scanning was performed using a LG-FS80 digital slide scanner (Servicebio, Wuhan, China). All specimens were evaluated in a double-blinded manner by two experienced pathologists. Immunostaining was initially assessed holistically at low magnification (×100), followed by averaging the scores from five randomly selected fields at higher magnifications (×200 or ×400). Expression levels of positive cells were semiquantitatively scored using the Histochemical score (H-score), which integrates staining intensity and the proportion of positive cells. In brief, the intensity of staining was scored as 0 (−), 1 (+), 2 (++), or 3 (+++), denoting no staining, light staining, intermediate staining, or dark staining, respectively, by visual assessment. The H-score was calculated based on the following formula: H = (1 × percentage of cells of light staining) + (2 × percentage of cells of intermediate staining) + (3 × percentage of cells of dark staining).

### 2.12 Animal models

All animal experimental procedures were approved by the Institutional Animal Care and Use Committee of Zhengzhou University. Six-week-old male BALB/c-nude mice were purchased from Beijing Vital River Laboratory Animal Technology (Beijing, China) and housed under specific pathogen-free conditions. To investigate whether the LAMP5 gene promotes tumor progression *in vivo*, 3 × 10^6^ SW480-shLAMP5 or HCT116-shLAMP5 cells were subcutaneously implanted into the right flank of BALB/c-nude mice.

### 2.13 Western blotting

Cells were lysed on ice for 30 min in RIPA lysis buffer (Beyotime, P0013B) supplemented with a protease inhibitor cocktail. The lysate was centrifuged at 12,000 rpm for 20 min at 4 °C, and the protein concentration in the supernatant was determined using a BCA protein assay kit (Beyotime, P0010). Equal amounts of protein (20 μg per lane) were separated by SDS-PAGE and transferred onto a PVDF membrane (Millipore, IPVH00010). The membrane was blocked with 5% (w/v) non-fat milk in TBST for 1 h at room temperature. After three washes with TBST, the membrane was incubated with primary antibodies (Supplementary Table S1) for 1 h at room temperature or overnight at 4 °C. Following three washes with TBST, the membrane was incubated with HRP-conjugated secondary antibodies (Supplementary Table S1) for 2 h at room temperature. After three final washes with TBST, protein bands were visualized using Super ECL Plus substrate (UElandy, S6009L) and imaged with a Tanon chemiluminescent imaging system (Tanon, Tanon-4600SF).

### 2.14 Statistical analysis

All bioinformatics analyses were performed using R software (v 4.4.2). Experimental data were analyzed using GraphPad Prism (v10.1.2). Differences between two groups were evaluated using Student’s t-test when appropriate, whereas comparisons among multiple groups were conducted using one-way analysis of variance or nonparametric alternatives as required. Kaplan-Meier survival analysis was performed using the log-rank test. Univariate Cox proportional hazards regression was used for the multi-cohort prognostic screening of subtype-associated genes. To assess the independent prognostic value of LAMP5, multivariate Cox regression adjusting for age, sex, and AJCC stage was additionally performed in the TCGA cohort, in which complete clinical annotation was available. For *in vivo* tumor growth curves, repeated-measures analysis was applied as appropriate. Unless otherwise specified, a two-sided P value <0.05 was considered statistically significant. Multiple testing correction was performed using the Benjamini–Hochberg method where applicable.

## 3 Results

### 3.1 Pathway-based consensus clustering identifies four pathway-based molecular subtypes of colorectal cancer

**FIGURE 1:**
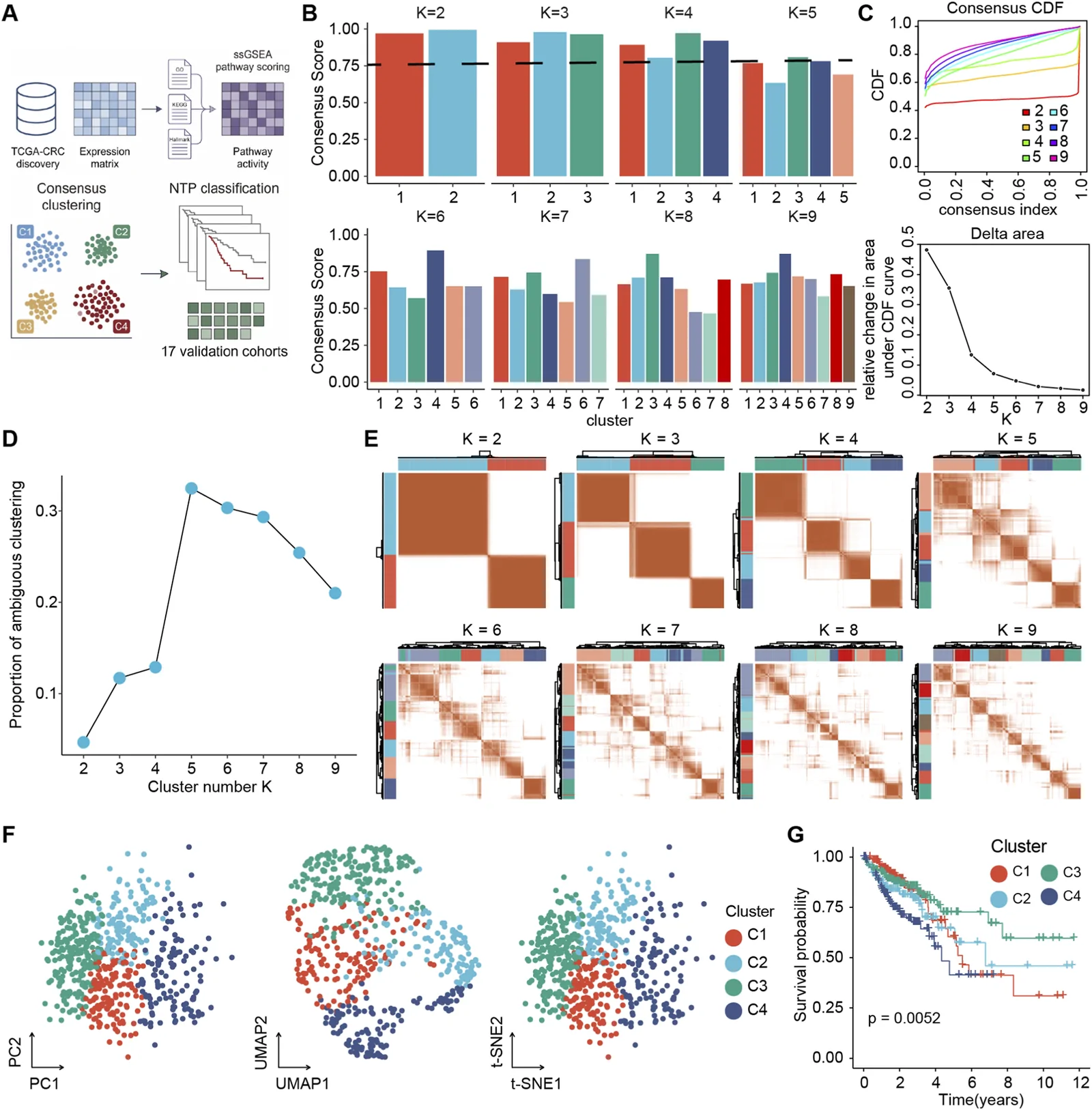
Identification of pathway-based molecular subtypes of colorectal cancer. **(A)** Overview of the analytical workflow, from TCGA-CRC pathway activity scoring and consensus clustering to nearest template prediction (NTP)-based classification across 17 validation cohorts. **(B)** Cluster consensus scores across candidate cluster numbers (K = 2–9). **(C)** Consensus cumulative distribution function (CDF) curves and the corresponding delta area plot. **(D)** Proportion of ambiguous clustering (PAC) across K. **(E)** Consensus matrices for K = 2 to K = 9. **(F)** PCA, UMAP, and t-SNE of pathway activity profiles colored by subtype (C1-C4). **(G)** Kaplan-Meier overall survival of the four subtypes (log-rank P = 0.0052).

To define pathway-based molecular subtypes of CRC, we first calculated pathway activity scores for TCGA CRC samples using gene sets from the GO, KEGG, and Hallmark collections (Figure 1A). Based on the resulting pathway activity matrix, consensus clustering was performed to identify a stable subtype structure. Evaluation of clustering stability across candidate cluster numbers showed that K = 4 was the optimal solution, as supported by the cumulative distribution function curves, delta area plot, and proportion of ambiguous clustering analysis (Figures 1B–D). The consensus matrix at K = 4 displayed a clear block-diagonal pattern with high within-cluster consistency and low between-cluster overlap, indicating robust separation of the four subtype groups (Figure 1E).

To further assess the distinctness of these subtype assignments, we performed PCA, UMAP, and t-SNE on the pathway activity profiles. All three dimensionality reduction approaches consistently demonstrated clear separation among the four clusters, designated C1, C2, C3, and C4 (Figure 1F). Survival analysis further showed that these subtypes were clinically relevant. Kaplan-Meier analysis revealed a significant difference in overall survival among the four groups (P = 0.0052), with patients in the C3 subtype exhibiting the most favorable prognosis and those in the C4 subtype showing the poorest outcome (Figure 1G). These findings indicate that pathway-based clustering can stratify CRC into four biologically distinct subtypes and identify a high-risk subtype associated with inferior survival.

### 3.2 External validation confirms the robustness of the four-subtype framework and reveals distinct functional programs

**FIGURE 2:**
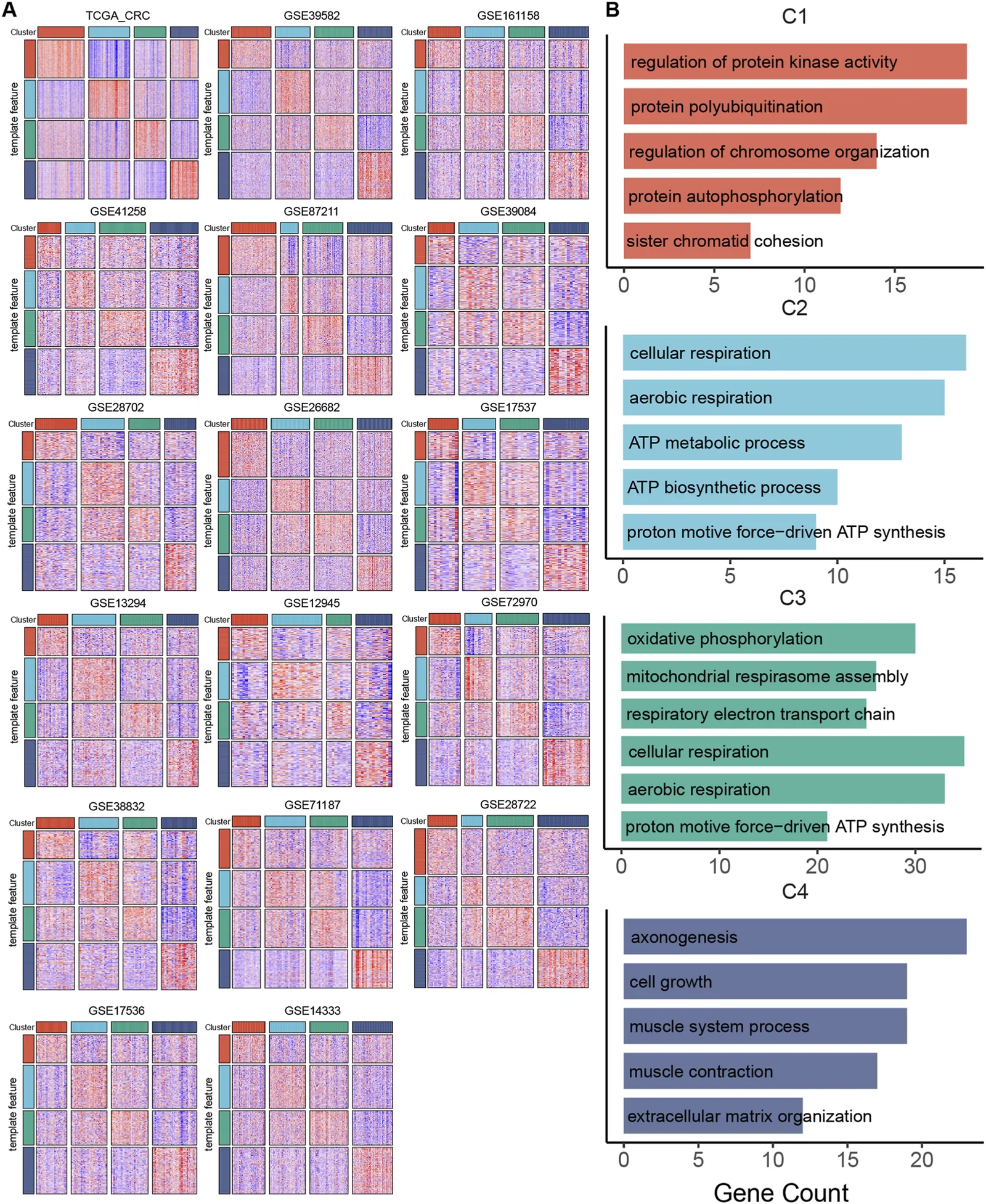
External validation and functional characterization of the four-subtype framework. **(A)** Nearest template prediction (NTP) classification of the four pathway-based molecular subtypes across 17 independent CRC cohorts (heatmaps of template features by sample). **(B)** Gene Ontology biological process enrichment for each subtype (C1-C4; bar length = gene count).

To determine whether the pathway-based molecular subtype classification was reproducible beyond the discovery cohort, we applied NTP to 17 independent CRC cohorts and found a consistent four-group pattern (Figure 2A). These findings support the robustness and transferability of the pathway-based molecular subtype framework across independent CRC cohorts. We next investigated the biological characteristics associated with each subtype by performing Gene Ontology biological process enrichment analysis. The results showed that the four subtypes were associated with distinct functional programs (Figure 2B). Specifically, C1 was mainly enriched in processes related to regulation of protein kinase activity, protein polyubiquitination, chromosome organization, protein autophosphorylation, and sister chromatid cohesion, suggesting a proliferation-related phenotype. C2 and C3 were both characterized by enrichment in energy metabolism-related pathways, including cellular respiration, aerobic respiration, ATP metabolic process, ATP biosynthesis, oxidative phosphorylation, and respiratory electron transport, indicating metabolically active states. In contrast, C4 was enriched in extracellular matrix organization, cell growth, muscle system process, muscle contraction, and axonogenesis, consistent with a stromal remodeling-associated program. Together, these results demonstrate that the four-subtype model is reproducible across multiple CRC cohorts and captures biologically distinct states, with C4 showing features suggestive of a structurally remodeled and potentially more aggressive tumor phenotype.

### 3.3 Single-cell transcriptomic analysis maps the aggressive subtype-associated phenotype at cellular resolution

**FIGURE 3:**
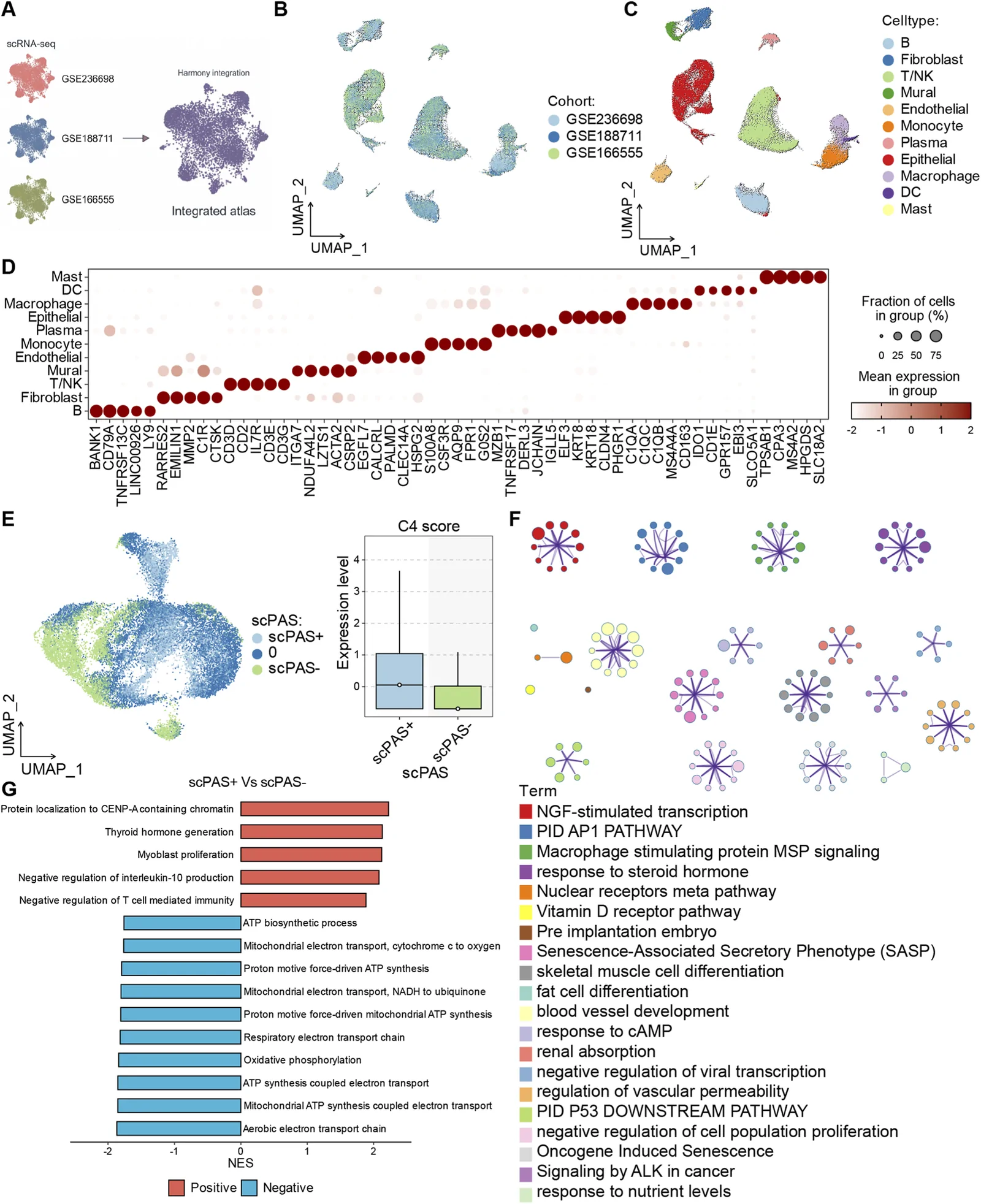
Single-cell transcriptomic mapping of the aggressive subtype-associated phenotype. **(A)** UMAP of three CRC scRNA-seq datasets (GSE236698, GSE188711, GSE166555) before integration. **(B)** UMAP after Harmony integration colored by dataset. **(C)** UMAP of the 11 annotated cell populations. **(D)** Dot plot of canonical marker genes used for cell-type annotation (dot size = fraction of expressing cells; color = mean expression). **(E)** UMAP colored by scPAS group (scPAS+, neutral, scPAS-) and C4-associated scores in scPAS+ versus scPAS- cells. **(F)** Cell population-level enrichment of signaling programs, including the senescence-associated secretory phenotype (SASP), macrophage-stimulating protein (MSP) signaling, p53 downstream signaling, and oncogene-induced senescence. **(G)** GSEA comparing scPAS+ and scPAS- cells (positive = enriched in scPAS+; negative = enriched in scPAS-).

To investigate the cellular basis of the aggressive pathway-based molecular subtype, we integrated three independent CRC scRNA-seq datasets (GSE236698, GSE188711, and GSE166555) using the Harmony algorithm (Figures 3A,B). Unsupervised clustering followed by manual annotation based on canonical marker genes identified 11 major cell populations, including B cells, fibroblasts, T/NK cells, mural cells, endothelial cells, monocytes, plasma cells, epithelial cells, macrophages, dendritic cells, and mast cells (Figures 3C,D). This integrated atlas provided the basis for mapping the bulk-derived subtype signal onto individual cells. To project the C4-associated phenotype to single-cell resolution, we calculated a single-cell pathway activity score using the top 300 C4-related genes and classified cells into scPAS+, neutral, and scPAS− groups. Cells in the scPAS+ group exhibited significantly higher C4-associated scores than those in the scPAS− group, supporting the presence of aggressive bulk-defined subtype-associated signals at the single-cell level (Figure 3E). Functional enrichment analysis further showed that multiple signaling programs differed across cell populations, including pathways related to senescence-associated secretory phenotype, macrophage stimulating protein signaling, p53 downstream signaling, and oncogene-induced senescence (Figure 3F). In addition, gene set enrichment analysis comparing scPAS+ and scPAS− cells showed that scPAS+ cells were enriched in processes associated with chromatin organization and immune regulation, whereas scPAS− cells were enriched in oxidative phosphorylation, mitochondrial electron transport, and ATP biosynthetic processes (Figure 3G). These findings suggest that the aggressive subtype is associated with a distinct cellular phenotype characterized by altered immune-associated activity and reduced mitochondrial metabolic features.

### 3.4 Multi-cohort prognostic screening identifies LAMP5 as the top unfavorable prognostic gene in colorectal cancer

**FIGURE 4:**
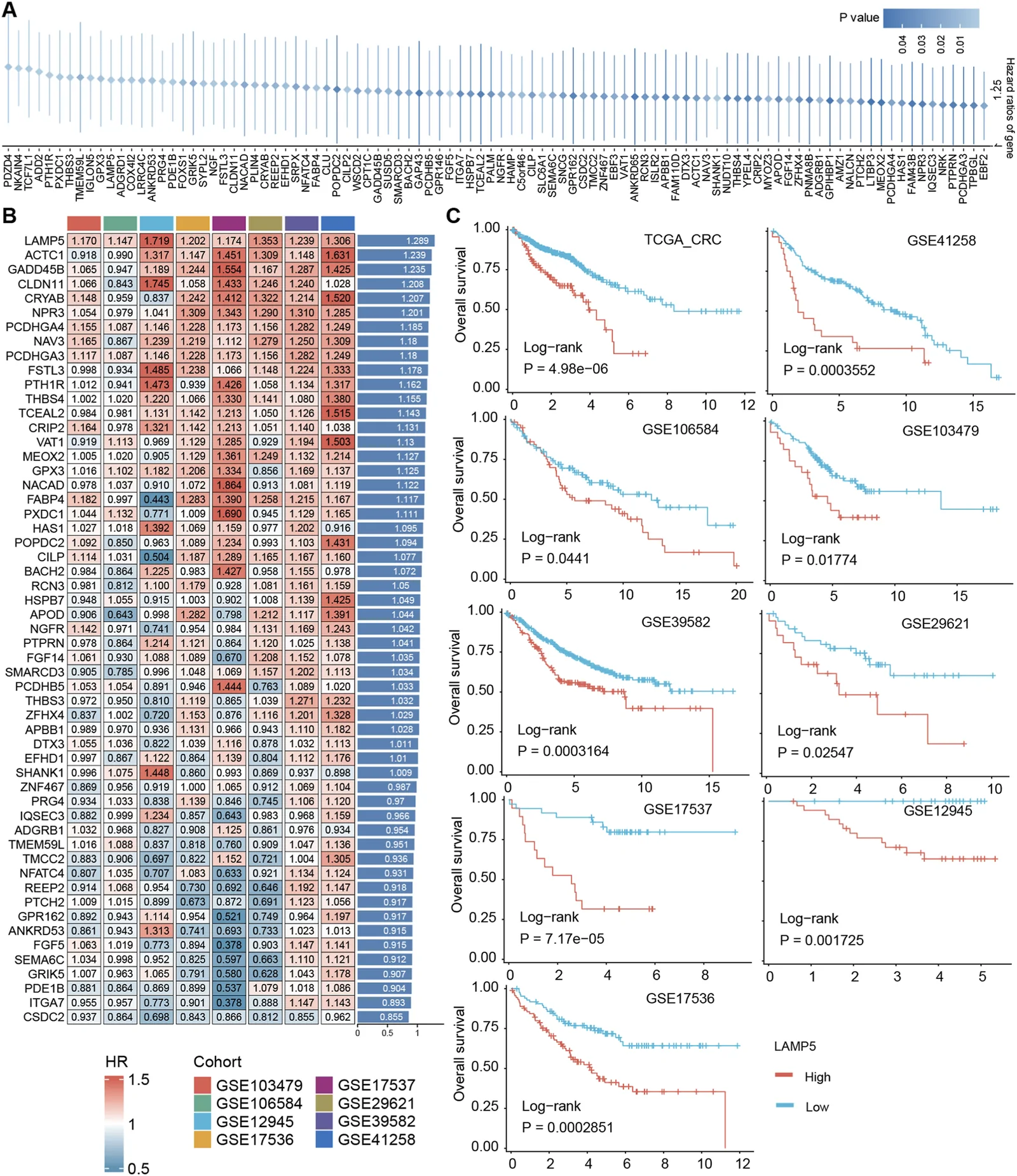
Multi-cohort prognostic screening identifies LAMP5 as the top unfavorable prognostic gene in colorectal cancer. **(A)** Distribution of univariate Cox hazard ratios for the 300 subtype-discriminative genes across TCGA-CRC and GEO cohorts (dots colored by P value). **(B)** Heatmap of hazard ratios for candidate genes across cohorts; LAMP5 ranked first, with hazard ratios greater than one in all datasets. **(C)** Kaplan-Meier overall survival for high versus low LAMP5 expression in nine independent CRC cohorts (log-rank test).

After characterizing the aggressive subtype in bulk data and mapping its associated signal at the single-cell level, we next sought to identify subtype-associated genes with consistent prognostic significance across independent CRC cohorts. Using the top 300 subtype-discriminative genes as candidate features, we performed univariate Cox proportional hazards regression in TCGA-CRC and eight GEO validation cohorts. Comparison of hazard ratio distributions across datasets showed marked heterogeneity among candidate genes, but a subset displayed stable prognostic direction across cohorts (Figures 4A,B). Among these genes, LAMP5 emerged as the most consistent unfavorable prognostic factor, ranking first based on cross-cohort reproducibility and showing hazard ratios greater than one in all analyzed datasets (Figure 4B). To further validate the prognostic relevance of LAMP5, Kaplan-Meier survival analysis was performed in nine independent CRC cohorts. In every dataset, patients with high LAMP5 expression had significantly shorter overall survival than those with low expression (Figure 4C). In multivariate Cox regression adjusting for age, sex, and AJCC stage in the TCGA cohort, high LAMP5 expression remained independently associated with poorer overall survival, indicating that its prognostic value is not merely a reflection of conventional clinicopathological staging. These findings identify LAMP5 as a reproducible poor-prognosis gene among the subtype-associated candidates and support its prioritization for further biological characterization.

### 3.5 LAMP5 expression is associated with advanced clinicopathological progression and aggressive subtype-related cellular states

**FIGURE 5:**
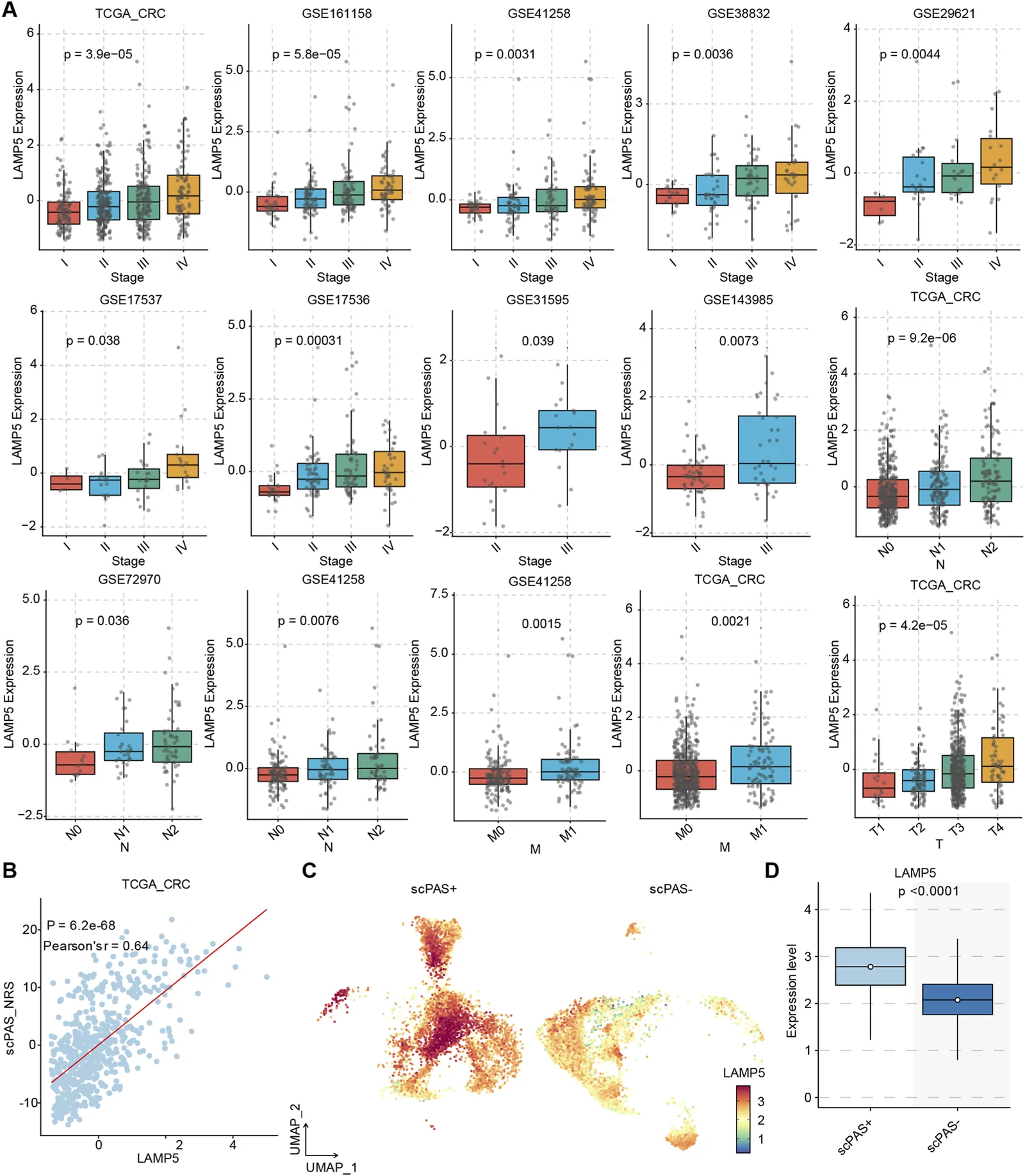
LAMP5 expression is associated with advanced clinicopathological progression and aggressive subtype-related cell states. **(A)** LAMP5 expression across AJCC stage, T stage, N stage, and M stage in multiple independent CRC cohorts. **(B)** Correlation between LAMP5 expression and the single-cell pathway activity score (scPAS) normalized risk score in TCGA-CRC (Pearson r = 0.64). **(C)** UMAP of single cells colored by LAMP5 expression in scPAS+ and scPAS- populations. **(D)** LAMP5 expression in scPAS+ versus scPAS- cells (P < 0.0001).

To evaluate the clinical relevance of LAMP5, we next examined its association with clinicopathological progression in multiple CRC cohorts. LAMP5 expression increased with advancing AJCC stage in nine independent datasets and also showed positive associations with more advanced N stage in three datasets, M stage in two datasets, and T stage in the TCGA cohort (Figure 5A). These results indicate that elevated LAMP5 expression is associated not only with poor survival but also with more advanced disease progression. We then assessed whether LAMP5 was linked to the aggressive subtype-associated transcriptional state. In TCGA_CRC, LAMP5 expression was strongly positively correlated with the scPAS normalized risk score (r = 0.64, P < 0.001), indicating a close relationship between LAMP5 and the phenotype associated with the aggressive pathway-based molecular subtype (Figure 5B). Consistent with this finding, single-cell analysis showed that LAMP5 expression was significantly enriched in scPAS+ cells compared with scPAS− cells (P < 0.0001) (Figures 5C,D). Together, these data indicate that LAMP5 is tightly associated with clinicopathological progression and preferentially localized to cell populations carrying the aggressive subtype signal.

### 3.6 LAMP5 is linked to TGFβ-EMT signaling, and experimental validation supports its pro-tumorigenic role in colorectal cancer

**FIGURE 6:**
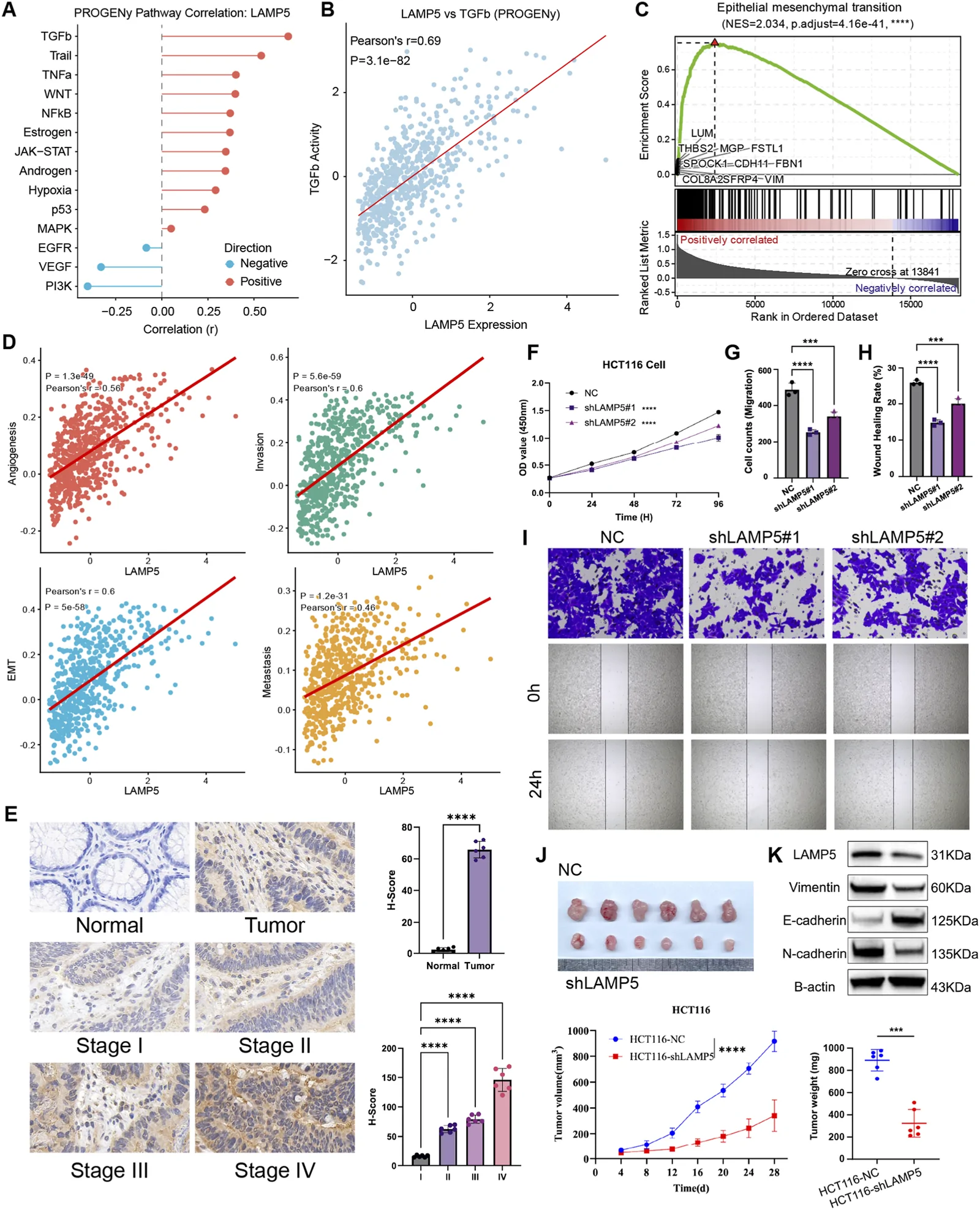
LAMP5 is linked to TGFβ-EMT signaling and promotes malignant progression *in vitro* and *in vivo*. **(A)** PROGENy pathway activity correlation with LAMP5 (lollipop plot; red = positive, blue = negative). **(B)** Correlation between LAMP5 expression and TGFβ pathway activity (Pearson r = 0.69). **(C)** GSEA enrichment plot for the Hallmark epithelial-mesenchymal transition (EMT) gene set (NES = 2.034, adjusted P < 0.001). **(D)** Correlation of LAMP5 expression with ssGSEA scores for angiogenesis, invasion, EMT, and metastasis programs. **(E)** Representative immunohistochemistry (IHC) staining of LAMP5 in normal versus tumor tissue and across Stage I-IV, with H-score quantification (****P < 0.0001). **(F)** CCK-8 proliferation assay in HCT116 cells after LAMP5 knockdown (NC, shLAMP5#1, shLAMP5#2). **(G)** Quantification of Transwell migration (cell counts). **(H)** Quantification of wound-healing migration (%). **(I)** Representative images of Transwell migration and wound healing (0 h and 24 h). **(J)** Xenograft assay showing representative tumors, tumor growth curves, and tumor weights (HCT116-NC versus HCT116-shLAMP5). **(K)** Western blot of LAMP5, Vimentin, E-cadherin, N-cadherin, and β-actin after LAMP5 knockdown.

To explore the signaling programs associated with LAMP5, we performed pathway activity inference using PROGENy in TCGA-CRC. LAMP5 expression was positively correlated with 12 of 14 inferred pathways, with the strongest association observed for TGFβ signaling (r = 0.69, P < 0.001) (Figures 6A,B). Given the established role of TGFβ in epithelial-mesenchymal transition and metastatic progression, we next assessed whether LAMP5 was associated with EMT-related transcriptional programs (Figure 6C). Gene set enrichment analysis showed that samples with higher LAMP5-associated gene expression were significantly enriched for the Hallmark EMT pathway (NES = 2.034, adjusted P < 0.001), with leading-edge genes including THBS2, MGP, FSTL1, CDH11, FBN1, and VIM. In addition, ssGSEA revealed that LAMP5 expression was positively correlated with hallmark programs related to angiogenesis (r = 0.56), invasion (r = 0.50), EMT (r = 0.60), and metastasis (r = 0.46) (Figure 6D). These results consistently indicate that LAMP5 is embedded in a transcriptional program associated with mesenchymal transition and malignant progression. IHC staining showed that LAMP5 expression was elevated in tumor tissues compared with normal controls and progressively increased from Stage I to Stage IV (Figure 6E). In HCT116 cells, LAMP5 knockdown suppressed proliferation (CCK-8, Figure 6F), reduced migration (Transwell, Figure 6G; wound healing; Figure 6H; representative images; Figure 6I), and decreased xenograft tumor growth (Figure 6J). Furthermore, LAMP5 silencing upregulated E-cadherin and downregulated N-cadherin and Vimentin (Figure 6K). These findings support a role for LAMP5 in the malignant progression of colorectal cancer.

## 4 Discussion

In the present study, we established a pathway-based molecular classification framework for colorectal cancer and identified four biologically distinct subtypes with different prognostic outcomes. Among them, the C4 subtype consistently exhibited the poorest survival and was characterized by extracellular matrix remodeling- and stromal-associated programs. This four-subtype framework was reproducible across 17 independent CRC cohorts, supporting its robustness across datasets. By integrating bulk transcriptomic analysis with single-cell RNA sequencing data, our analysis further suggested that the aggressive subtype-associated signal could be projected to specific cellular states using scPAS. Since multi-cohort prognostic screening, LAMP5 emerged as the most consistent unfavorable prognostic gene among subtype-associated candidates. Additional clinicopathological analyses, pathway inference, and experimental validation collectively indicated that LAMP5 is closely associated with CRC progression and linked to EMT signaling.

Current molecular classification systems such as CMS and CRIS have substantially advanced the understanding of CRC heterogeneity, but they are still primarily based on gene-expression patterns (Isella et al., 2017; Oropeza-Martínez et al., 2025). In contrast, the present study adopted a pathway-centered strategy, which captures coordinated biological programs rather than relying solely on individual transcript abundance (Zhang et al., 2025a). This distinction may be meaningful for at least two reasons. First, pathway-level features can improve biological interpretability by linking subtype structure directly to functional states. Second, they may be less sensitive to cohort-specific noise and technical variation than gene-level signatures. In our analysis, the four-subtype model showed stable transferability across 17 independent datasets, supporting the reproducibility of this strategy. Notably, the poor-prognosis C4 subtype was enriched for stromal remodeling and extracellular matrix-related programs, a pattern that is conceptually consistent with the mesenchymal and unfavorable features reported in CMS4, although the present framework was derived from pathway activity rather than conventional transcriptome clustering (Hänzelmann et al., 2013). These findings suggest that pathway-based subtyping can provide a complementary perspective on CRC heterogeneity and may improve the functional interpretation of aggressive disease states.

The adverse outcome of the C4 subtype may be partly attributed to stromal activation and extracellular matrix remodeling. Extracellular matrix remodeling alters tissue architecture and enhances tumor cell–matrix interactions, thereby facilitating local invasion and metastatic dissemination. Stromal activation and TGFβ-related signaling are also closely associated with EMT-related plasticity and invasive behavior. In addition, stromal remodeling can contribute to an immunosuppressive tumor microenvironment and weaken antitumor immune surveillance. Collectively, these features provide a plausible biological explanation for the aggressive phenotype and poor prognosis of the C4 subtype.

One of the major findings of this study is the identification of LAMP5 as a robust adverse prognostic factor in CRC. LAMP5 belongs to the lysosome-associated membrane protein family, whose members have been implicated in membrane trafficking, lysosomal regulation, autophagy, and tumor biology (Martinez-Romero et al., 2018). Compared with other members of this family, LAMP5 has received relatively limited attention. Nevertheless, previous evidence suggests that it may participate in malignant progression in several tumor types. Umeda et al. reported that LAMP5 promotes the metastatic potential of gastric cancer cells, supporting a role in aggressive tumor behavior (Umeda et al., 2022). Other studies have linked LAMP5 to oncogenic processes in hematologic malignancies and additional solid tumors. In the current study, LAMP5 showed hazard ratios greater than one across all analyzed cohorts and remained significantly associated with poor overall survival in nine independent datasets, indicating remarkable consistency as an unfavorable prognostic marker. Moreover, LAMP5 expression increased with advancing clinicopathological stage and was enriched in scPAS+ cells, further supporting its association with aggressive biological states.

Our pathway analyses further suggest that the biological role of LAMP5 in CRC may be closely linked to TGFβ-EMT signaling. Among the inferred pathways, TGFβ showed the strongest correlation with LAMP5 expression, and GSEA identified significant enrichment of the Hallmark EMT program in LAMP5-associated transcriptional profiles (Wu et al., 2024; Lin et al., 2026). Additional ssGSEA results linked LAMP5 to angiogenesis, invasion, EMT, and metastasis-related programs. These observations are biologically plausible in the context of CRC progression, as TGFβ signaling is a well-established regulator of epithelial-mesenchymal plasticity, stromal activation, immune remodeling, and metastatic dissemination (Tao et al., 2024). EMT is widely recognized as a central process in CRC invasion and dissemination (Li et al., 2024), and TGFβ-mediated EMT has been repeatedly implicated in advanced disease and poor outcome. Our results therefore place LAMP5 within a broader transcriptional and signaling context associated with mesenchymal transition and tumor progression. Importantly, this interpretation was supported by functional experiments showing that LAMP5 knockdown suppressed proliferation, migration, and invasion *in vitro* and reduced xenograft tumor growth *in vivo*, with accompanying changes in EMT-related markers. Taken together, these findings support the view that LAMP5 is not merely a passive prognostic correlate but may actively contribute to aggressive CRC behavior. However, the present findings do not establish a direct regulatory effect of LAMP5 on TGFβ signaling, and further studies involving SMAD phosphorylation, TGFβ stimulation, and rescue experiments are needed to clarify this relationship.

Another important aspect of this study is the integration of bulk subtype analysis with single-cell transcriptomic mapping. Although bulk molecular subtypes have been widely used in CRC research, their direct biological interpretation at the cellular level is often limited. By applying scPAS to integrated scRNA-seq datasets, we were able to project the aggressive C4-associated program to individual cells and identify scPAS+ populations enriched for this phenotype. This approach adds an additional layer of interpretability to the subtype framework by showing that aggressive transcriptional programs are not simply cohort-level abstractions but can be localized to distinct cellular states within the tumor microenvironment. The enrichment of immune-regulatory and chromatin-associated programs in scPAS+ cells, together with the relative depletion of oxidative phosphorylation-related programs, suggests that the aggressive subtype may be linked to a reprogrammed tumor ecosystem characterized by altered immune activity and metabolic adaptation. Thus, the combination of pathway-based subtyping and single-cell projection provides a useful framework for connecting patient-level prognosis with cell-state biology.

From a translational and pharmacological perspective, our findings suggest that LAMP5 may have value both as a prognostic biomarker and as a molecule with therapeutic exploratory value. Its stable association with poor survival across multiple cohorts indicates that LAMP5 may help identify high-risk CRC patients who could be prioritized for more intensive monitoring or treatment. More importantly, the strong association between LAMP5 and TGFβ-EMT signaling places LAMP5 within a signaling axis that is itself an active pharmacological target. TGFβ-pathway-directed agents, including small-molecule TGFβ receptor kinase inhibitors (for example, galunisertib and vactosertib) and TGFβ-neutralizing or bifunctional biologics, are being evaluated in colorectal and other cancers (Tao et al., 2024; Tang et al., 2026). Because mesenchymal, TGFβ-activated CRC—conceptually related to the poor-prognosis C4 subtype identified here—is frequently associated with stromal immune exclusion and limited response to immune checkpoint blockade, a LAMP5-high, TGFβ-EMT-activated transcriptional state may help define the patient subset most likely to benefit from TGFβ-targeted therapy, or from combinations of TGFβ blockade with immunomodulatory agents. In this context, LAMP5 could serve as a candidate companion biomarker for stratifying patients in studies of anti-cancer immunomodulators, where therapeutic efficacy is increasingly recognized to depend on tumor cell state and microenvironmental composition. Although these possibilities remain hypothesis-generating and require dedicated pharmacological and preclinical validation, they illustrate how the present mechanistic findings could directly inform future intervention and patient-selection strategies. Accordingly, additional work is required before clinical translation, and the present results support further investigation of LAMP5 in biomarker development and in mechanistic studies focused on invasive CRC phenotypes.

Several limitations of this study should be acknowledged. First, the bioinformatics analyses were based largely on retrospective public datasets, which may contain cohort-specific biases that cannot be fully eliminated. Second, although our experimental results support a tumor-promoting role for LAMP5, the direct molecular mechanism linking LAMP5 to TGFβ signaling and EMT remains to be clarified. Third, although consistent effects of LAMP5 knockdown were observed in both SW480 and HCT116 cells, gain-of-function experiments were not performed in the present study and should be addressed in future work. Finally, prospective clinical studies will be necessary to determine whether LAMP5 can be incorporated into routine prognostic assessment or therapeutic decision-making. Despite these limitations, the consistent findings across pathway-based clustering, external validation, single-cell mapping, prognostic screening, and functional experiments provide convergent support for the biological and clinical relevance of LAMP5 in CRC.

In summary, this study established a pathway-based molecular subtyping framework for CRC and identified C4 as a poor-prognosis subtype characterized by stromal remodeling-related programs. Through multi-cohort analysis and single-cell projection, we further identified LAMP5 as a robust unfavorable prognostic biomarker associated with aggressive tumor states. Integrated computational and experimental evidence linked LAMP5 to the TGFβ-EMT axis and supported its role in CRC progression. These findings provide a basis for improved biological stratification of CRC and suggest that LAMP5 may represent a promising candidate for future prognostic and therapeutic investigation.

## 5 Conclusion

This study established a pathway-based molecular classification framework for colorectal cancer and identified four biologically distinct subtypes with different clinical and functional characteristics. Among them, the C4 subtype consistently showed the poorest prognosis and was enriched in stromal remodeling-related programs. By integrating cross-cohort validation, single-cell projection, prognostic screening, and experimental verification, we identified LAMP5 as a robust unfavorable prognostic biomarker associated with aggressive tumor states. At the molecular level, LAMP5 was closely linked to TGFβ signaling, epithelial-mesenchymal transition, invasion, and metastasis-related programs, and functional assays supported its tumor-promoting role in CRC. These findings provide a framework for improved molecular stratification of CRC and suggest that LAMP5 may represent a promising candidate for future prognostic and therapeutic investigation.

## Data Availability

The original contributions presented in the study are included in the article/Supplementary Material, further inquiries can be directed to the corresponding author.
